# Bioluminescent reporter assay for monitoring ER stress in human beta cells

**DOI:** 10.1038/s41598-018-36142-4

**Published:** 2018-12-10

**Authors:** Maria J. L. Kracht, Eelco J. P. de Koning, Rob C. Hoeben, Bart O. Roep, Arnaud Zaldumbide

**Affiliations:** 1 Departments of Cell and Chemical Biology, Leiden University Medical Center, Leiden, The Netherlands; 2 Departments of Immunohematology & Blood Transfusion, Leiden University Medical Center, Leiden, The Netherlands; 3 Internal Medicine, Leiden University Medical Center, Leiden, The Netherlands; 4 Department of Diabetes Immunology, Diabetes & Metabolism Research Institute, City of Hope, Duarte, USA

## Abstract

During type 1 diabetes development, cells in the islets of Langerhans engage adaptive mechanisms in response to inflammatory signals to cope with stress, to restore cellular homeostasis, and to preserve cell function. Disruption of these mechanisms may induce the formation of a repertoire of stress-induced neoantigens, which are critical in the loss of tolerance to beta cells and the development of autoimmunity. While multiple lines of evidence argue for a critical role of the endoplasmic reticulum in these processes, the lack of tools to specifically monitor beta cell stress hampers the development of therapeutic interventions focusing on maintaining endoplasmic reticulum homeostasis. Here we designed and evaluated a stress-induced reporter in which induction of stress correlates with increased light emission. This Gaussia luciferase-based reporter system employs the unconventional cytoplasmic splicing of XBP1 to report ER stress in cells exposed to known ER-stress inducers. Linking this reporter to a human beta cell-specific promotor allows tracing ER-stress in isolated human beta cells as well as in the EndoC-βH1 cell line. This reporter system represents a valuable tool to assess ER stress in human beta cells and may aid the identification of novel therapeutics that can prevent beta cell stress in human pancreatic islets.

## Introduction

Beta cell destruction in Type 1 diabetes (T1D) results from the combined effect of inflammation and autoimmunity. The presence of endoplasmic reticulum (ER) stress markers during insulitis points to the involvement of an ER stress response in beta cell destruction^1^. The ER is a central organelle for protein synthesis, processing and folding and essential in insulin biosynthesis, maturation and secretion^2,3^. Perturbations of the ER homeostasis by environmental factors triggers the induction of an unfolded protein response (UPR) and activation of inositol-requiring protein 1α (IRE1α), protein kinase RNA-like endoplasmic reticulum kinase (PERK) and the cleavage of membrane bound activating transcription factor 6 (ATF6). Activation of these ER membrane bound sensors leads to phosphorylation of eukaryotic translation initiation factor 2α by PERK^4^, activation of transcription factor X-box binding protein 1 (XBP1) via nonconventional XBP1 RNA splicing by IRE1α^5,6^ and translocation of ATF6 to the nucleus^7^, respectively. These different pathways ultimately act in concert to restore ER homeostasis by the coordinated regulation of inhibition of protein synthesis, degradation of aberrant translation products by the ERAD degradation pathway and enhanced protein folding capacity by upregulation of chaperone expression.

The high insulin translation rate makes beta cells extremely sensitive to ER stress^8^ and several lines of evidence suggest that the UPR is a key mechanism for the formation of neoantigens and subsequent autoimmune destruction of beta cells^9–11^. We and others have shown that pathophysiological conditions characteristic for T1D participate to the increased complexity of the beta cell proteome by affecting alternative splicing events^12^, formation of defective ribosomal products (DRiPs)^13^, activation of post translational modification enzymes leading to citrullination and deamidation of autoantigens^14–18^. These processes are likely to increase visibility of beta cells to immune cells and their subsequent destruction. Thus, monitoring and understanding the origin of beta cell stress is critical to understand autoimmunity, to prevent beta cell failure and to design therapeutics to prevent T1D development. While quantitative methods for monitoring ER stress *in vitro* are well established, these methods are labour intensive and cannot be translated to selectively address beta cell stress in human pancreatic islets because of their multi-endocrine nature. In this study, we describe a quantitative bioluminescent method to measure ER stress by exploiting the UPR-induced IRE1α-mediated splicing of XBP1 coupled to a Gaussia luciferase reporter gene. We show that this reporter accurately reflects the ER stress status in the human beta cell line EndoC-βH1 during inflammation when compared to classical ER stress quantification methods. Furthermore, it can be used to specifically monitor beta cell stress in primary human islets when the reporter expression is driven by the human insulin promoter (HIP). This reporter represents a novel tool to identify therapeutics targeting beta cell stress in a drug screening platform in human beta cells.

## Results

### Design of ER stress reporter

**Figure 1:**
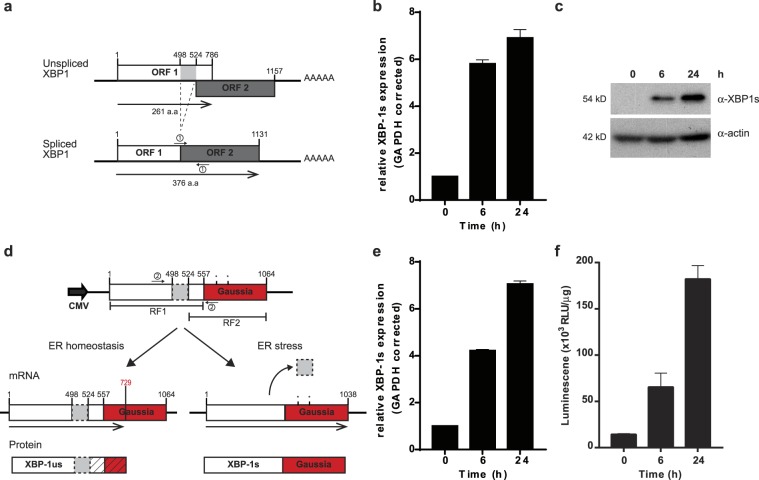
Principle of the ER stress reporter. (**a**) Schematic representation of XBP1 mRNA under normal (top) and ER stress conditions (bottom). XBP1 mRNA consists of two open reading frames (ORFs) (white and dark grey box). In normal conditions, the retained intron (light grey box) limits translation to the first ORF. Upon ER stress, the intronic region is spliced out by p-IRE1α as indicated by the dashed lines, allowing a shift of the first ORF, to the second ORF generating a protein with an extended C-terminus. The functional ORF is indicated by the black arrow underneath. Position of the primer set for analysis of spliced XBP1 are indicated with small arrows with the number 1. (**b**) Quantification of XBP1s by qPCR in 293 T cells after exposure to TG for 0, 6 or 24 hours. Quantitative PCR analysis was performed in triplicate and GAPDH corrected. (**c**) Western blot analysis of 293 T cell lysates treated with 100 nM TG for 0, 6 or 24 hours analysed with an anti-XBP1-spliced antibody (upper panel) and anti-actin antibody (lower panel). Unprocessed images are presented in supplementary Figure 1. (**d**) Schematic representation of the ER stress reporter construct, pLV-CMV-XBP-GLuc-bc-Puro, encoding XBP1 (white box) with retained intron (light grey box) and Gaussia luciferase (red box) driven by a CMV promoter. The two ORFs are indicated by black lines. In normal conditions, the intronic region is retained and only the first ORF is translated (left arm). The splicing of XBP1 during ER stress results in the translation of the first and second ORF resulting in an XBP1-Gaussia fusion protein (right arm). In red the in-frame STOP codon of the first ORF is depicted. The diagonal line pattern indicates the translational product is not in the functional reading frame. Position of the primer set for analysis of reporter splicing are indicated with small arrows with the number 2. (**e**) Quantification of XBP1s by qPCR in cells transfected with the reporter construct after exposure to TG for 0, 6 or 24 hours. Quantitative PCR analysis was performed in triplicate and GAPDH corrected. (**f)** Luciferase activity measured in pLV-CMV-XBP-GLuc-bc-Puro transfected 293 T cell lysates priorly treated with 100 nM TG for 0, 6 or 24 hours. Results are shown as relative light units (RLU) corrected by the protein amount in the sample.

Activation of the IRE1 endonuclease by ER stress leads to unconventional XBP1 splicing in which a 26 nucleotide intronic region is removed (Fig. 1a,b). This process causes a shift of the reading frame and gives rise to translation of an elongated C-terminal protein as observed by Western blot analysis of lysates from 293 T cells exposed to ER stress-inducing agent thapsigargin (TG) (Fig. 1c). We exploited this stress-induced splicing mechanism to generate a lentivirus vector containing a stress-inducible Gaussia luciferase reporter (pLV-CMV-XBP-GLuc-bc-Puro) (Fig. 1d). In this bi-cistronic construct, where the puromycin resistant gene can be used for clone selection, the ER stress-dependent splicing positioned the Gaussia luciferase coding sequence in frame with the XBP1 *bona-fide* AUG to generate a XBP-Gaussia luciferase fusion protein. Following transfection in HEK 293 T cells with the XBP-GLuc construct, treatment with TG lead to up to increase XBP1 splicing as detected by mRNA analysis (Fig. 1e) and to 10-fold induction in light emission after 24 h treatment, compared to untreated cells, indicating that the reporter is induced by ER stress (Fig. 1f).

### Validation and characterization of the ER stress reporter in 293 T cells

**Figure 2:**
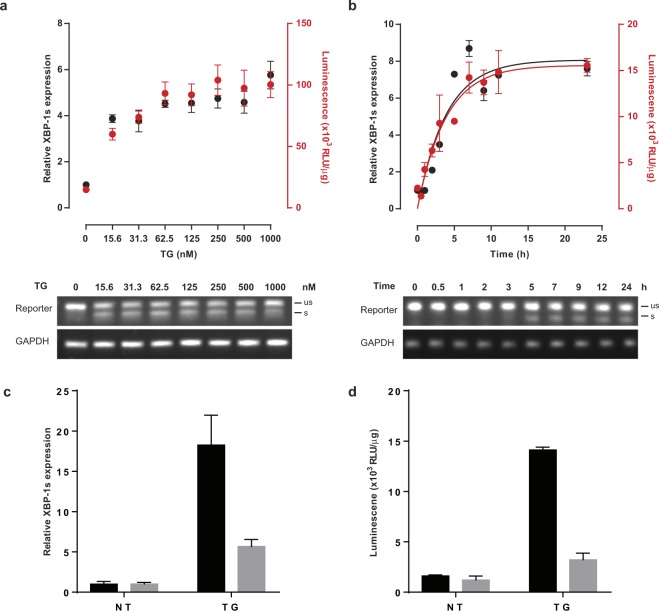
Characterization of pLV-CMV-XBP-GLuc-bc-Puro in 293 T cells. (**a**) Analysis of the ER stress-induced reporter in transfected 293 T cells exposed to various amounts of TG by classic validation of endogenous XBP-1 splicing by qPCR (black dots) and luciferase activity (red dots) (upper panel); Validation of reporter splicing by PCR analysis of reporter and GAPDH (lower panel) on agarose gel. (**b**) Similarly, a time course analysis of the ER-stress reporter in transfected 293 T cells stimulated with 50 nM TG was performed. Quantitative PCR analysis was performed in triplicate and GAPDH corrected. Gaussia activity is expressed in relative light units per μg protein. Each point represents the average of 3 experimental replicates. Data are shown as the mean ± SEM. Amplification curves belonging to the qPCR data are presented in supplementary Figure 2. (**c**) qPCR analysis of the level of expression of XBP1s in 293 T cells transfected with the reporter construct after 24 hours treatment with 100 nM TG in presence or absence of 5 µM MKC3946. Quantitative PCR analysis was performed in triplicate and GAPDH corrected. (**d**) Luciferase activity measured in transfected 293 T cell lysates after 24 hours treatment with 100 nM TG in presence or absence of 5 µM MKC3946. Results are shown as relative light units (RLU) corrected by the protein amount in the sample.

To test the sensitivity of the stress reporter in more detail, 293 T cells were transfected with pLV-CMV-XBP-GLuc and exposed to various amounts of TG for 6 hours or to a moderate amount of stress agent and analysed over a 24-hour period. ER stress was evaluated by i) the conventional ER stress quantification method, qPCR using primers specific for spliced XBP1 (Fig. 2a,b black symbols); and ii) light emission after cell lysis to quantify reporter splicing (Fig. 2a,b red symbols). In these experiments, a similar spectrum of ER stress quantification was obtained with both ER stress quantification methods. Using both techniques we show that XBP1 splicing is occurring at minimal amounts of TG, shortly after exposure to stress inducer and rapidly reaching a plateau when higher doses are used or 8 hours post stimulation. Light emission correlated strongly with the splicing of the reporter construct as determined by PCR using primers specific for the reporter (Fig. 2a,b lower panels). To evaluate whether the reporter construct may be used as drug screening assay, we tested the effect of the MKC3946^19^, an inhibitor of inositol-requiring enzyme 1α in HEK293T cells in presence of ER stress inducer TG. As anticipated, the induction of stress by TG lead to a substantial increase in XBP1s RNA expression. This effect was countered by the presence of the MKC3946, indicating effective inhibition of XBP1 splicing (Fig. 2c). Similar results were obtained by measuring Gaussia luciferase activity in the cell lysates (Fig. 2d).

Altogether, these data indicate that the reporter accurately reflects stress-induced splicing of endogenous XBP-1, even shortly after exposure to low amount of stress inducing agent and allows screening of ER stress inhibitor drugs.

### Evaluation of the ER stress reporter in human beta cells

**Figure 3:**
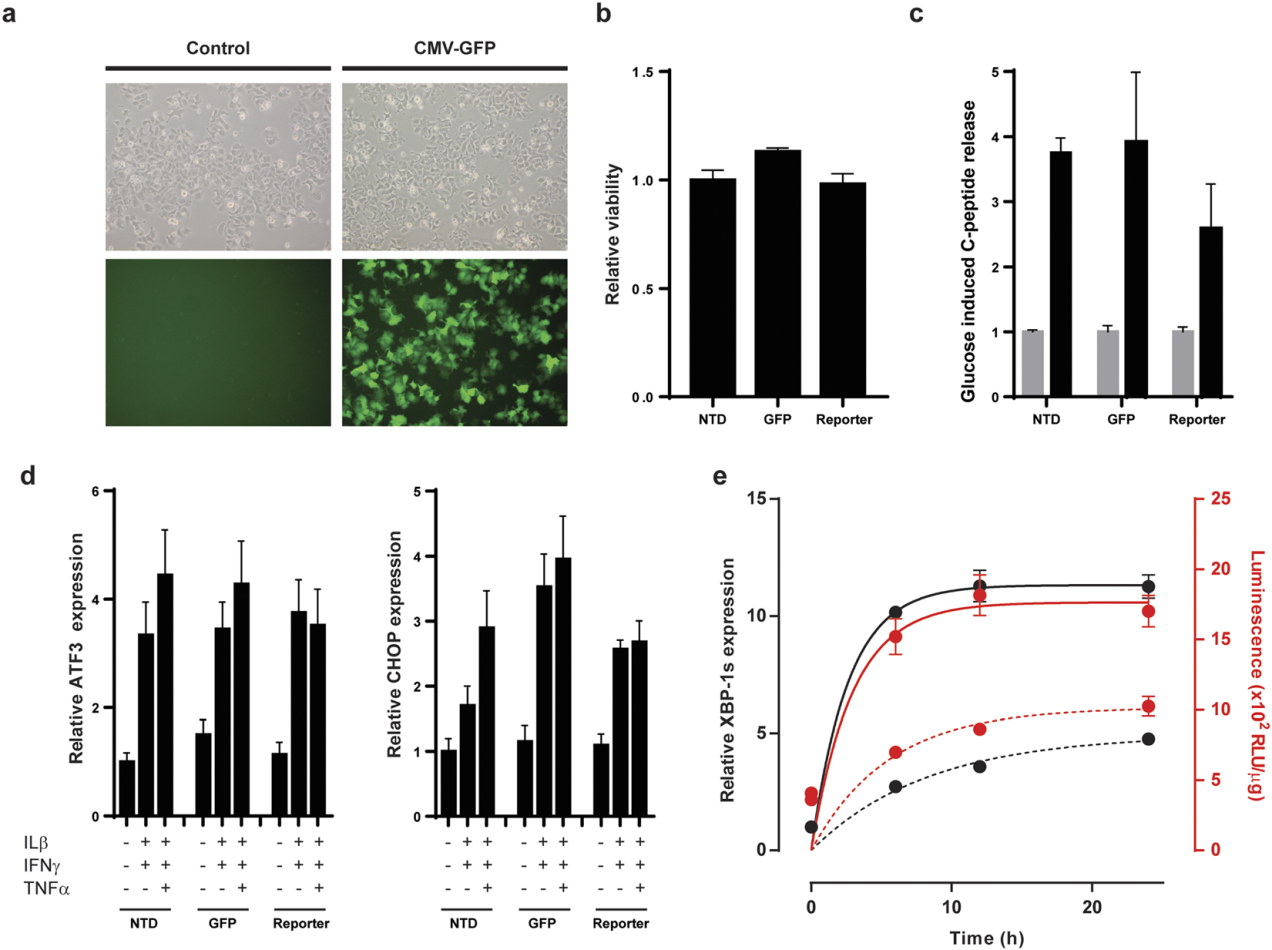
Evaluation of the ER stress reporter in human beta cells. (**a**) Transduction of EndoC-βH1 with LV-CMV-GFP after puromycin selection (right panels) compared to non-transduced cells (left panels). (**b**) Cell viability as determined by WST assay in non-transduced cells (NTD), GFP transduced (GFP) or transduced with the pLV-CMV-XBP-GLuc-bc-Puro construct (reporter). Data are shown as relative viability compared to non-transduced cells (set to 1). (**c**) C-peptide release assay performed on non-transduced cells (NTD), GFP transduced (GFP) or the reporter cell lines (reporter) after low glucose stimulation (grey bars) or high glucose stimulation (black bars). Data are shown as induction c-peptide release, low glucose conditions set to 1. (**d**) qPCR analysis of the level of expression of ATF3 and CHOP in non-transduced cells (NTD), GFP transduced (GFP) or the reporter cell lines (reporter) after cytokines treatment. Quantitative PCR analysis was performed in triplicate and GAPDH corrected. The data are shown as relative expression. The expression in non-transduced cells in absence of stimulation is set to 1 and used as reference. (**e**) Analysis of the reporter EndoC-βH1 cells exposed to 1000 U/ml IFNγ and 2 ng/ml IL-1β (dashed lines) and 1000 U/ml IFNγ, 2 ng/ml IL-1β and 50 ng/ml TNFα (solid lines) for 0, 6, 12 and 24 hours. ER stress was validated using endogenous XBP-1 splicing by qPCR (black lines and dots) and luciferase activity (red lines and dots).

Beta cell stress is a significant component underlying the pathogenesis of T1D. Recently, the human EndoC-βH1 beta cell line has been generated as a promising model to study human beta cell biology under T1D pathophysiological conditions^20^. In order to evaluate the potential of the XBP-GLuc construct to detect ER stress in a relevant beta cell model, we generated a stable reporter cell line by lentiviral transduction followed by puromycin selection; pLV-CMV-GFP-bc-Puro transduced cells were used as control (Fig. 3a). We confirmed that lentiviral transduction and expression of the reporter construct had no apparent impact on beta cell viability (Fig. 3b) and function as seen by c-peptide secretion after glucose challenge (Fig. 3c). Moreover, the presence of the transgene had no effect on the baseline of UPR related gene expression and capacity to respond to stress stimulation as seen by the increased expression of ATF3 and CHOP upon cytokines stimulation (Fig. 3d). The modified cells were then exposed to proinflammatory cytokines mimicking the pathogenic inflammatory beta cell microenvironment of pancreatic islet in type 1 diabetes, i.e. IFNγ/IL1β or IFNγ/IL1β/TNFα and analysed for endogenous XBP1 splicing by qPCR or light emission in cell lysates. Both treatments led to the generation of endogenous XBP1s, which correlated with an increase in luminescence (Fig. 3e). Of note, the induction of stress is enhanced by the presence of TNFα in the cytokine mixture.

**Figure 4:**
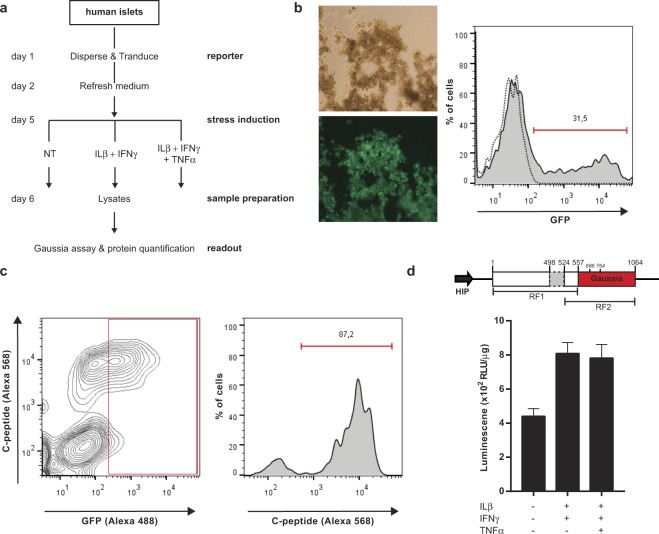
Evaluation of the ER stress reporter in primary human beta cells. (**a**) Flow diagram of the experimental design for the evaluation of beta cell stress in human pancreatic islets. (**b**) flow cytometry analysis showing the transduction rate of primary human islet cells after cell dispersion and lentivirus transduction with LV-CMV-GFP (MOI = 3). (**c**) Flow cytometry analysis of primary human islets cells stained with anti-c-peptide and anti-GFP antibodies after cell dispersion and transduction with LV-HIP-GFP (MOI = 3). (**d**) Schematic representation of the beta cell-specific ER stress reporter construct, encoding XBP1 (white box) with retained intron (light grey box) and Gaussia luciferase (yellow box) driven by a HIP promoter (black box arrow), and underneath the two reading frames (indicated by black lines) (top). Graph depicts the luminescence measured in lysates made of transduced human islets after exposure to inflammatory cytokines (1000 U/ml IFNγ, 2 ng/ml IL-1β and 50 ng/ml TNFα) (bottom). Each point represents the average of 3 experimental replicates. Data are shown as the mean ± SEM.

While classical methods allow a precise examination of the ER stress status, the presence of various cell types in the islets of Langerhans hampers the use of these methods for specific investigation of beta cells. In order to study beta cell stress within their islet environment we replaced the CMV promoter by the human insulin promoter (HIP) to drive expression of the transgene specifically in insulin-producing cells (Fig. 4a)^21^. We show that while lentivirus vectors can moderately modify endocrine cells (~30% transduction efficiency) (Fig. 4b), the use of the human insulin promoter restricts expression of the transgene to beta cells (Fig. 4c).

Following transduction with the pLV-HIP-XBP-GLuc-bc-GFP lentiviral vector, human endocrine cells were exposed to pro-inflammatory cytokines. Using this assay, we demonstrate that primary human beta cells respond to inflammatory cytokines by eliciting an ER stress response, as reflected by the increased Gaussia activity. Furthermore, our data point to a major contribution of IFNγ/IL1β in this process since no increase in light emission was detected upon addition of TNFα to the cytokine mixture (Fig. 4d).

## Discussion

Islets of Langerhans are responsible for maintaining glucose homeostasis. The ER forms the integration interface between the cellular microenvironment and adaptation. Dysfunction of this interface may contribute to the development of T1D by generation of neoantigens targeted by the immune system and by inducing cell death pathways. Therefore, the ability to monitor ER stress is an essential step in understanding disease pathogenesis and for the development of therapeutic intervention.

The Gaussia luciferase reporter assay presented here is an easy and rapid alternative to traditional methods. In contrast to previous approaches, the stress status can be directly measured in cell lysates upon addition of substrate, without the need of laborious RNA isolation or protein analysis procedures. The IRE1α-XBP1 pathway has a critical role in life and death decision by preserving ER homeostasis versus triggering apoptosis by activating the regulated IRE1-dependent decay (RIDD)^22^. In beta cells, the IRE1α-XBP1 pathway participates to the maintenance of the balance between the beta cell microenvironment and beta cell function as it is required for proper insulin biosynthesis and glucose responsiveness^23–25^. By exploiting XBP1 processing in the reporter construct, we demonstrate that this reporter accurately reflects endogenous XBP-1 splicing after both chemical- and cytokines-induced stress in 293T and EndoC-βH1 cells. This approach appeared equally sensitive as currently used ER stress detection methods. The generation of stable EndoC-βH1 cells expressing the ER stress reporter set the stage for future high throughput compound screening aiming at relapsing ER stress^20^ and can be combined with other assays to monitor beta cell functionality, such as the glucose stimulated insulin secretion (GSIS) assay.

The multi-endocrine nature of human pancreatic islets often complicates the analysis of beta cell stress in primary islets, as the classical methods do not distinguish which cells are stressed but rather provide a general picture of stressed islet cells as whole. This impairs assessing beta cell stress selectively, as the other endocrine cell may respond differently to changes in the microenvironment counteracting the beta cell stress^26,27^. Single cell analyses of pancreatic islets are currently the only method offering a real reflection of beta cell stress specifically. Yet, these techniques are difficultly translatable for drugs screening approach. The method described here, using the human insulin promoter to drive transgene expression of an ER stress reporter specifically in beta cells, represents a rapid and robust alternative allowing the specific analyses of beta cell ER stress within the total islet environment. Our results using the IRE1α inhibitor, on HEK293T cells show the potential application of the reporter as drug screening platform.

Our results indicate that EndoC-βH1 cells respond differently to cytokines when compared to human islets. The relative low induction of ER stress detected in primary beta cells compared to the beta cell line after cytokine stimulation, might be the result of a high basal level of ER stress in islets resulting from islet isolation, *in vitro* culture conditions and cell dispersion thereby dampening *in vitro* ER stress responses to cytokines. Moreover, EndoC-βH1 cells seem particularly sensitive to TNFα in the cytokine stimulation cocktail. Previous studies comparing the response of the human beta cell line and primary islets to cytokines, have shown that the EndoC-βH1 cells may have an impaired ER stress response and do not faithfully reflect the response of human islets to cytokines^28^. While differences in response to cytokines between EndoC-βH1 and primary human beta cells could reflect intrinsic differences between the cell line and primary cells (e.g. EndoC-βH1 cells divide while primary beta cells do not), these differences could also point towards a guardian role of other endocrine cells^27^. The glucagon like peptide 1 (GLP1) produced by alpha cells for example could represent a possible paracrine mediator of this anti-inflammatory effect^29^. Therefore, caution should be taken with the use of beta cell lines and, where feasible, results obtained from cell lines should be confirmed in primary human beta cells.

While similar strategies for ER stress evaluation have been successfully used in transgenic mice with a XBP1-GFP variant^30^, the combination of the lentivirus vector that can efficiently modify cells without affecting cell function^31^ and the Gaussia luciferase allow a sensitive and quantitative method to measure stress. We anticipate that this reporter used in combination with the human insulin promoter will represent a powerful tool to address various issues regarding ER stress in beta cells in type 1 diabetes as well as type 2 diabetes.

## Materials and Methods

### Cell culture

HEK293T cells were maintained in high glucose Dulbecco’s modified Eagle’s medium (DMEM, Gibco-BRL, Breda, The Netherlands) supplemented with 8% fetal bovine serum (FBS) (Gibco-BRL), 100 units/ml Penicillin and 100 μg/ml streptomycin (Gibco-BRL).

The human beta cell line, EndoC-βH1 cells^32^, was obtained from Dr. Raphael Scharfmann (Paris Descartes University, France) and maintained in low glucose DMEM supplemented with 5.5 μg/ml human transferrin, 10 mM Nicotinamide, 6.7 ng/ml Selenit, 50 μM β-mercaptoethanol, 2% bovine serum albumin fraction V, 100 units/ml Penicillin and 100 μg/ml streptomycin. Cells were seeded in ECM, fibronectin pre-coated culture plates. Inflammatory stress was induced by treating EndoC-βH1 with a mixture of 1000 U/ml IFNγ, 2 ng/ml IL1β and 50 ng/ml TNFα for the indicated time.

### Transfection

HEK293T were transfected in suspension using polyethylenimine (PEI). Transfection mixes, consisting of 0.125 μg plasmid DNA, 0.375 μg/μl PEI in a total volume of 12.5 μl Opti-MEM(GIBCO) for a 96-well, were incubated for 10 minutes at room temperature prior adding to the cell suspension. After 24 hours cells were treated with Thapsigargin (Bio-connect). The concentrations and incubation times are indicated in the figures. For treatment with the IRE1α inhibitor, transfected cells were pre-treated for 4 h with MKC3946 (5uM) before addition of 0.1uM Thapsigargin for 24 hours.

### Human islet isolation and culture

Pancreatic islets were obtained from human organ donor pancreata. Human islets were isolated from organ donors. Islets were only studied if they could not be used for clinical purposes and if research consent had been obtained. According to the national law ethics approval is not required for research on donor tissues that cannot be used for clinical transplantation. The isolations were performed in the GMP-facility of LUMC according to the previously described protocol^33^. For experimental use, human islets were maintained in ultra-low attachment plates (Corning, NY 14831) in low glucose DMEM supplemented with 10% FBS, 100 units/ml Penicillin and 100 μg/ml streptomycin. Dispersed islet cells were treated with 1000 U/ml IFNγ, 2 ng/ml IL1β and 50 ng/ml TNFα for 24 hours to induce ER stress. All methods were carried out in accordance with relevant guidelines and regulations.

### Lentivirus production and transduction

Lentiviruses were produced as previously described^31^. Prior transduction the human pancreatic islets were dispersed, then cocultured with virus supernatant supplemented with 8 μg/ml polybrene (Sigma). After overnight incubation, the medium was refreshed and the cells were allowed to rest for 3 days, before continuing with other treatments and analysis. EndoC-βH1 stably expressing the reporter construct were established after transduction with the pLV-CMV-XBP-GLuc-bc-Puro lentivirus (MOI = 5) and puromycin selection (1ug/ml).

Human islets cells have been transduced at MOI = 3 immediately after cell dispersion by trypsin as previously described^13,21^. After overnight incubation, the medium was refreshed and dispersed islet cells were maintained in culture in ultra-low attachment plate (Corning, NY 14831) for the duration of the experiment.

### Construction of a beta cell-specific, stress-induced reporter

The XBP-Gaussia fragment contains the first 558 nucleotides of XBP1 coding sequence fused to the last 504 nucleotides from the GLucM43I encoding sequence^34^. The DNA fragment was synthesized by Geneart (thermofisher, Regensburg Germany). pLV-CMV-XBP-GLuc-bc-Puro was generated by insertion of the XBP-Gaussia fragment into pLV-CMV vector. The XBP Gaussia fragment was then subcloned into the pLV-HIP vector^21^ to generate a pLV-HIP-XBP-GLuc-bc-GFP vector.

### RNA isolation and quantitative PCR (qPCR)

RNA was extracted using TRIzol reagent (invitrogen) according to manufacturer’s procedures. To avoid DNA contamination samples were DNase treated prior cDNA synthesis. RNA was reverse transcribed using SuperScript III Reverse Transcriptase (Invitrogen). SybrGreen master mix kit (Applied Biosystems) was used for detection of endogenous XBP1 splicing, ATF3, CHOP and GAPDH by qPCR with the following primers: XBP1s Fw 5′-CTGAGTCCGCAGCAGGTG-3′, XBP1s Rv 5′-GAGATGTTCTGGAGGGGTGA-3′; ATF3 Fw 5′-GTGCCGAAACAAGAAGAAGG-3′, ATF3 Rv 5′-TCTGAGCCTTCAGTTCAGCA-3′; CHOP Fw 5′- GACCTGCAAGAGGTCCTGTC-3′, CHOP Rv 5′- CTCCTCCTCAGTCAGCCAAG-3′; GAPDH Fw 5′-ACAGTCAGCCGCATCTTCTT-3′, GAPDH Rv 5′-AATGAAGGGGTCATTGATGG-3′. All measurements were performed in triplicate and GAPDH was used for normalization. The detection of reporter splicing was assessed by PCR using the following primers: Reporter Fw 5′-GAAGCCAAGGGGAATGAAG-3′, Reporter Rv 5′-CCGTGGTCGCGAAGTTGCTGG-3′. PCR reactions were analysed on 2% agarose gel.

### Western blot analysis

Cells were lysed in Tropix lysis mix (Applied Biosystems). Total protein content of the lysates was determined using Bradford reagent assay (Biorad, Veenendaal, The Netherlands). Samples were boiled with sample buffer (10% glycerol, 2% SDS, 50 mM Tris-HCl pH 6.8, 0.1% Bromophenol blue and 1% β-mercaptoethanol) and 50 μg protein was loaded per well of a 10% SDS-polyacrylamide gel. The proteins were subsequently transferred to Immobilon-P (Millipore, Etten-Leur, the Netherlands) and visualized by standard protocols with rabbit-anti-XBP1, specific for the spliced XBP1 form (1:1000; Biolegend POLY6195) and mouse-anti-actin (1:2000 C4; Millipore) and corresponding HRP-conjugated antibodies goat-anti-rabbit IgG (1:5000 sc-2004, Santa Cruz Biotechnology) and goat-anti-mouse IgG HRP (1:5000 sc-2005, Santa Cruz Biotechnology).

### Gaussia activity measurement

Gaussia activity was determined in cell lysates by luminometry after addition of 1 mM coelenterazine (Promega). Experiments were performed in triplicate for every condition. Results are corrected for the lysate protein content and represented as average Relative light units (RLU) per μg protein.

### Glucose induced C-peptide assay

EndoC-βH1 were seeded 50,000 cells per well in a 96 well plate. Two days post plating, cells were preincubated in a modified Krebs-Ringer Bicarbonate buffer (KRBH) containing 115 mmol/l NaCl, 5 mmol/l KCl, 24 mmol/l NaHCO_3_, 2.2 mmol/l CaCl_2_, 1 mmol/l MgCl_2_, 20 mmol/l HEPES, 2 g/l human serum albumin (Cealb, Sanquin, The Netherlands), pH 7.4 for 2 hours. After preincubation, the buffer was successively changed for 1 hour to KRBH with 2 mmol/l and 25 mmol/l glucose at 37 °C. C-peptide concentrations were determined in the supernatants by ELISA (10-1136-01 Mercodia, Uppsala, Sweden).

### C-peptide staining

Following dispersion, single cell islet cells were fixed and permeabilized in PBS containing 4% paraformaldehyde and 0,1% saponin. Blocking was done with 1%BSA,0,1% saponin in PBS and in which primary and secondary antibodies were also diluted. Primary antibodies against C-peptide (1:1000; Millipore CBL94) and GFP (1:1000) and secondary antibodies coupled to Alexa 568 (1:500) and Alexa 488 (1:500) were used.

### Viability assay

Cell viability was assessed by replacing the culture medium by fresh medium containing 10% WST-1 reagent (Roche, Penzburg, Germany). The cells were maintained in the incubator at 37  C, 5% CO_2_ for 3 h before the absorbance was measured in a microplate reader (Bio-Rad model 550, Bio-Rad, Hercules, CA, USA) at a wavelength of 450 nm. The results are shown as relative viability compared to non-transduced cells.

### Statistical analysis

Data are presented as mean ± SEM. Trend lines were generated by nonlinear regression analysis following a one phase exponential association equation. Calculations were performed using GraphPad Prism 7.

## Electronic supplementary material

Supplementary information
