# Supplementary material for: Bioluminescent reporter assay for monitoring ER stress in human beta cells
Source: Sci Rep. 2018 Dec 10;8:17738. doi: 10.1038/s41598-018-36142-4 (PMC6288136; doi:10.1038/s41598-018-36142-4)
Supplement: Supplementary file 1 — Supplementary information [file 41598_2018_36142_MOESM1_ESM.pdf]

Supplementary Information:

Bioluminescent reporter assay for monitoring ER stress in human beta cells; Maria J.L. Kracht, Eelco J.P. de Koning, Rob C. Hoeben, Bart O. Roep and Arnaud Zaldumbide.

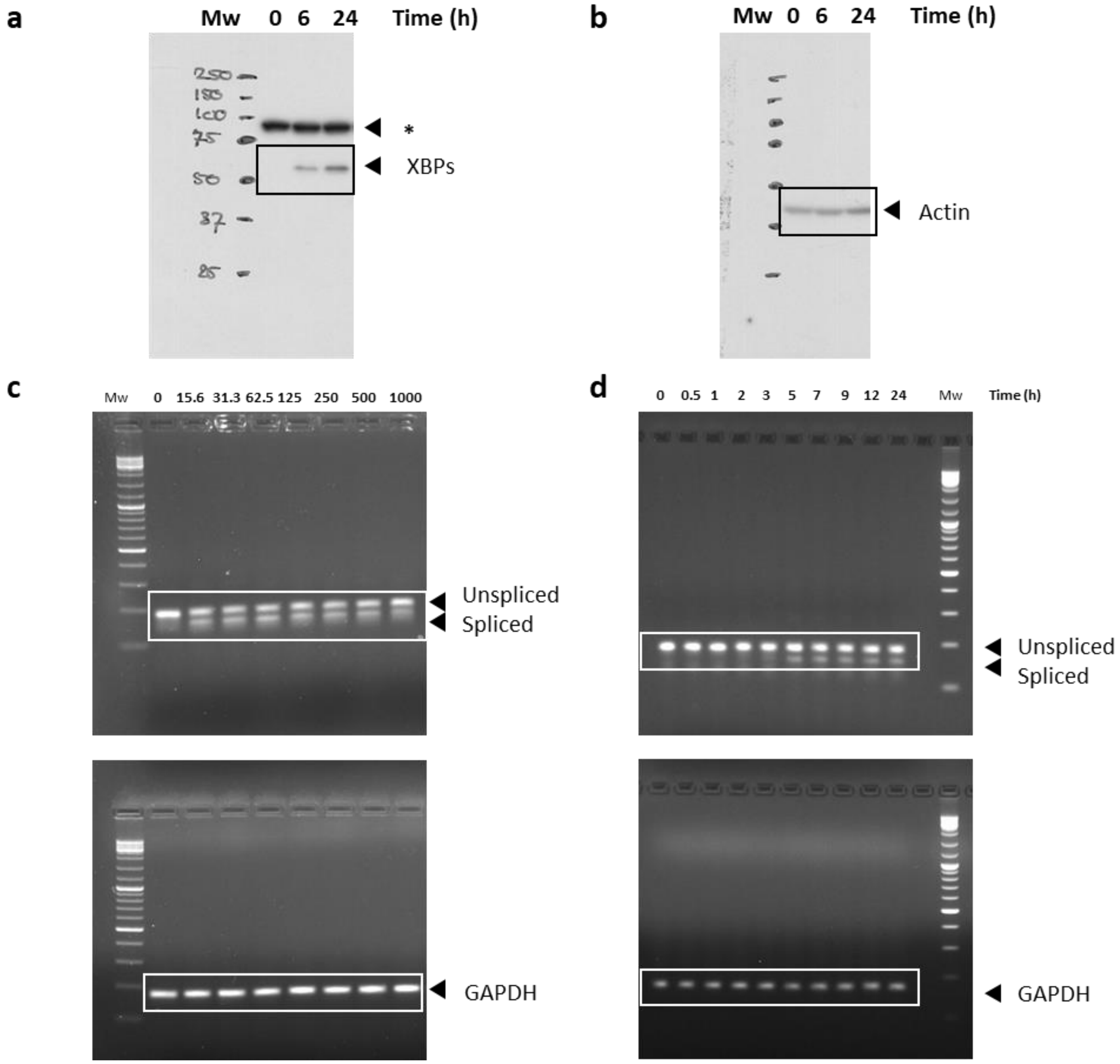

Supplementary figure 1: Unprocessed images presented in figure 1 and 2. a,b) Original picture for figure 1b showing the results of XBP staining (a), and actin staining (b). * indicates a non-specific band. c) Original picture for figure 2b showing the PCR results of samples analysed with XBP-reporter primers (primer set 2 as presented in figure 1a) (upper panel) and GAPDH (lower panel). d) c) Original picture for figure 2c showing the PCR results of samples analysed with XBP primers (upper panel) and GAPDH (lower panel). Boxes indicate the areas as depicted in the original figures. Mw indicates the molecular weight.

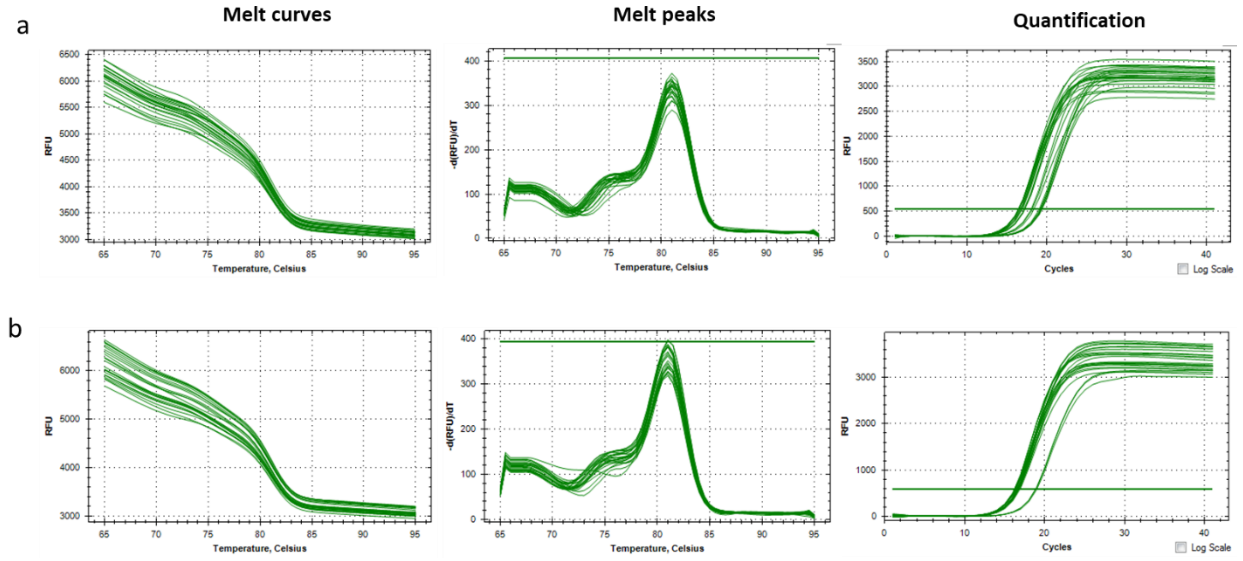

Supplementary figure 2: Amplification curves from qPCR using XBPs specific primers presented in figure 2. (a) Melt curves, melt peaks and quantification curves of the graph presented in figure 2a. (b) Melt curves, melt peaks and quantification curves of the graph presented in figure 2b.
